# Age-Dependent Control of Collagen-Dependent Platelet Responses by Thrombospondin-1—Comparative Analysis of Platelets from Neonates, Children, Adolescents, and Adults

**DOI:** 10.3390/ijms22094883

**Published:** 2021-05-05

**Authors:** Katrin Herken, Martin Glauner, Stefanie C. Robert, Matthias Maas, Sonja Zippel, Ulrike Nowak-Göttl, Barbara Zieger, Judith Lahav, Anke C. Fender, Kerstin Jurk, Beate E. Kehrel

**Affiliations:** 1 Department of Anaesthesiology, Intensive Care and Pain Medicine, Experimental and Clinical Haemostasis, University Hospital of Münster, 48149 Münster, Germany; katrin.herken@praxis-herken.de (K.H.); martin.glauner@pentapharm.com (M.G.); stefanie.robert@marien-kh-gmbh.de (S.C.R.); matthias.maas@fachklinik-hornheide.de (M.M.); sonja.zippel@gmx.de (S.Z.); Anke.Fender@uk-essen.de (A.C.F.); 2 DSM Nutritional Products, Branch Pentapharm, 4147 Aesch, Switzerland; 3 Fachklinik Hornheide, Department for Anaesthesiology, Intensive Care and Pain Medicine, 48157 Münster, Germany; 4 Department of Paediatric Haematology/Oncology, University of Münster, 48149 Münster, Germany; leagottl@uksh.de; 5 Institute of Clinical Chemistry, University Hospital Kiel-Lübeck, 24118 Kiel, Germany; 6 Department of Pediatrics and Adolescent Medicine, Division of Pediatric Hematology and Oncology, Medical Center, Faculty of Medicine, University of Freiburg, 79106 Freiburg, Germany; barbara.zieger@uniklinik-freiburg.de; 7 Hemostasis Laboratory, Rabin Medical Center-Beilinson Hospital, 49100 Petah-Tikva, Israel; judith.lahav@gmail.com; 8 Department of Human Genetics and Biochemistry, Sackler School of Medicine, Tel Aviv University, 39040 Tel Aviv, Israel; 9 Institute of Pharmacology, West German Heart and Vascular Center, University of Duisburg-Essen, 45147 Essen, Germany; 10 Center for Thrombosis and Hemostasis (CTH), University Medical Center, Johannes Gutenberg-University Mainz, 55131 Mainz, Germany

**Keywords:** neonates, platelets, collagen, thrombospondin-1, flow cytometry, reference ranges

## Abstract

Platelet function is developmentally regulated. Healthy neonates do not spontaneously bleed, but their platelets are hypo-reactive to several agonists. The mechanisms underlying immature platelet function in neonates are incompletely understood. This critical issue remains challenging for the establishment of age-specific reference ranges. In this study, we evaluated platelet reactivity of five pediatric age categories, ranging from healthy full-term neonates up to adolescents (11–18 years) in comparison to healthy adults (>18 years) by flow cytometry. We confirmed that platelet hypo-reactivity detected by fibrinogen binding, P-selectin, and CD63 surface expression was most pronounced in neonates compared to other pediatric age groups. However, maturation of platelet responsiveness varied with age, agonist, and activation marker. In contrast to TRAP and ADP, collagen-induced platelet activation was nearly absent in neonates. Granule secretion markedly remained impaired at least up to 10 years of age compared to adults. We show for the first time that neonatal platelets are deficient in thrombospondin-1, and exogenous platelet-derived thrombospondin-1 allows platelet responsiveness to collagen. Platelets from all pediatric age groups normally responded to the C-terminal thrombospondin-1 peptide RFYVVMWK. Thus, thrombospondin-1 deficiency of neonatal platelets might contribute to the relatively impaired response to collagen, and platelet-derived thrombospondin-1 may control distinct collagen-induced platelet responses.

## 1. Introduction

Platelets are first observed within 6 weeks of gestation, and numbers increase progressively to reach near-adult levels by week 22 of gestation [1]. Neonates show greater numbers of megakaryocyte and megakaryocyte progenitors with higher proliferative rates. In a subset of neonates, increased megakaryocyte polyploidy has been linked to larger and more reactive platelets and early-onset thrombocytopenia [2,3]. The special characteristics of neonatal megakaryocytes and platelets have been reviewed extensively [4,5,6]. Although healthy full-term neonates do not suffer from spontaneous hemorrhage, neonatal platelets show a relative hypo-functionality, characterized by subdued agonist-triggered signaling and activation responses [7,8]. This is largely associated with decreased dense body numbers [9], and the amount of cargo released [10,11]. Reduced platelet reactivity is also attributed to incomplete functional maturation, and this is particularly pronounced in pre-term neonates [12,13,14], accounting for the susceptibility of this subpopulation to intracranial hemorrhage [15,16].

There is some evidence that healthy children over 1 year of age show platelet function comparable to that of adults [17,18], but the time point when adult potential really is reached has not been clearly defined [19,20,21]. Moreover, the degree to which neonatal platelet reactivity to classical agonists is reduced compared to adults is highly variable due to pre-analytical variations and is critically dependent on the platelet function testing procedure [4,22].

Classically, platelet-vessel wall interactions are mediated by the subendothelial matrix protein collagen, and the plasma protein von Willebrand factor (VWF) exposed on the subendothelial collagen matrix and activated endothelial cells [23,24]. Previous studies in neonatal platelets have clearly shown comparatively lower collagen-induced aggregation [19,25,26] and Ca^2+^ mobilization [27]. This is mediated through the GPVI/FcRγ-chain and immunoreceptor tyrosine-based activation motif (ITAM)/Syk-downstream signaling as well as by the GPIa [28,29]. Others have demonstrated hypo-responsiveness of pre-term and full-term neonatal platelets to the GPVI-agonist collagen-related peptide, as detected by integrin αIIbβ3 activation, P-selectin surface expression [30] and Syk and PLCγ2 tyrosine phosphorylation, together with mildly reduced expression of GPVI but not of GPIa [31]. Conversely, adhesion and aggregation of neonatal platelets on a subendothelial extracellular matrix under arterial shear conditions are increased compared to adults [32]. Furthermore, neonatal platelets exhibit a shortened in vitro bleeding time measured by the platelet function analyzer devices in response to collagen/adenosine diphosphate (ADP) and collagen/epinephrine [33,34], demonstrating that higher levels of unusually large VWF multimers in the blood of neonates [35,36] contribute to the compensation for platelet hypo-reactivity to collagen and other agonists.

Previously, we identified the homotrimeric multidomain and matricellular glycoprotein thrombospondin-1 (TSP-1) as a high shear platelet substrate comparable to VWF, requiring GPIbα of the platelet GPIb/IX/V complex and to some extent the platelet TSP-1 receptor CD36 [37]. TSP-1 is synthesized by megakaryocytes and represents one of the most abundant proteins in platelet α-granules. Upon platelet activation, TSP-1 is secreted via α-granule exocytosis and promotes collagen-induced platelet aggregation via binding to CD36 [38]. Several years ago, we observed a defect in platelet response to collagen in a case of partial GPIa deficiency and platelet TSP-1 proteolytic cleavage that was resolved in vitro with exogenously added TSP-1 [39]. Synthetic peptides based on a sequence within the C-terminal cell-binding domain of TSP-1 have been shown to induce platelet agglutination via the integrin-associated protein (IAP, CD47) [40] and platelet activation in a FcRγ-chain-dependent manner [41]. Furthermore, TSP-1 mediates platelet interactions with inflamed vascular endothelial cells under flow [42] and with peripheral blood monocytes in patients with severe carotid artery disease [43]. However, the role of TSP-1 in the platelet function of neonates is unknown so far.

In this study, we systematically established platelet reactivity in up to five pediatric age categories, from healthy full-term neonates up to adolescents (11–18 years) in comparison to healthy adults (>18 years). Using flow cytometry, we assessed crucial platelet functions, i.e., fibrinogen binding (a marker of integrin αIIbβ3 activation), P-selectin surface expression (a marker of α-granule exocytosis), and CD63 surface expression (marker of δ-granule and lysosome exocytosis), in response to increasing concentrations of the platelet agonists TRAP-6, ADP and type I collagen to determine age-specific differences of platelet responsiveness. We hypothesize that platelet-derived TSP-1 plays a crucial role in the regulation of collagen-mediated platelet activation.

## 2. Results

### 2.1. Platelets from Neonates and Infants Are Differentially Hypo-Reactive After PAR-1 Stimulation

Neonatal platelets have been described to show diminished functional responses induced by several agonists compared to adult platelets. Here, we compared agonist-induced reactivity of platelets from five pediatric groups, ranging from full-term neonates to adolescents, to investigate age-dependent platelet reactivity patterns and to establish reference ranges assessed by flow cytometry. Three critical platelet activation responses, namely activation of the integrin α_IIb_β_3_ detected by surface binding of fibrinogen-FITC, α-granule exocytosis detected by CD62P/P-selectin surface expression, and exocytosis of δ-granules and lysosomes detected by CD63 surface expression [44,45], were measured in the different pediatric groups and normalized to the maximal response obtained with adult control platelets (>18 years) measured in parallel.

**Figure 1:**
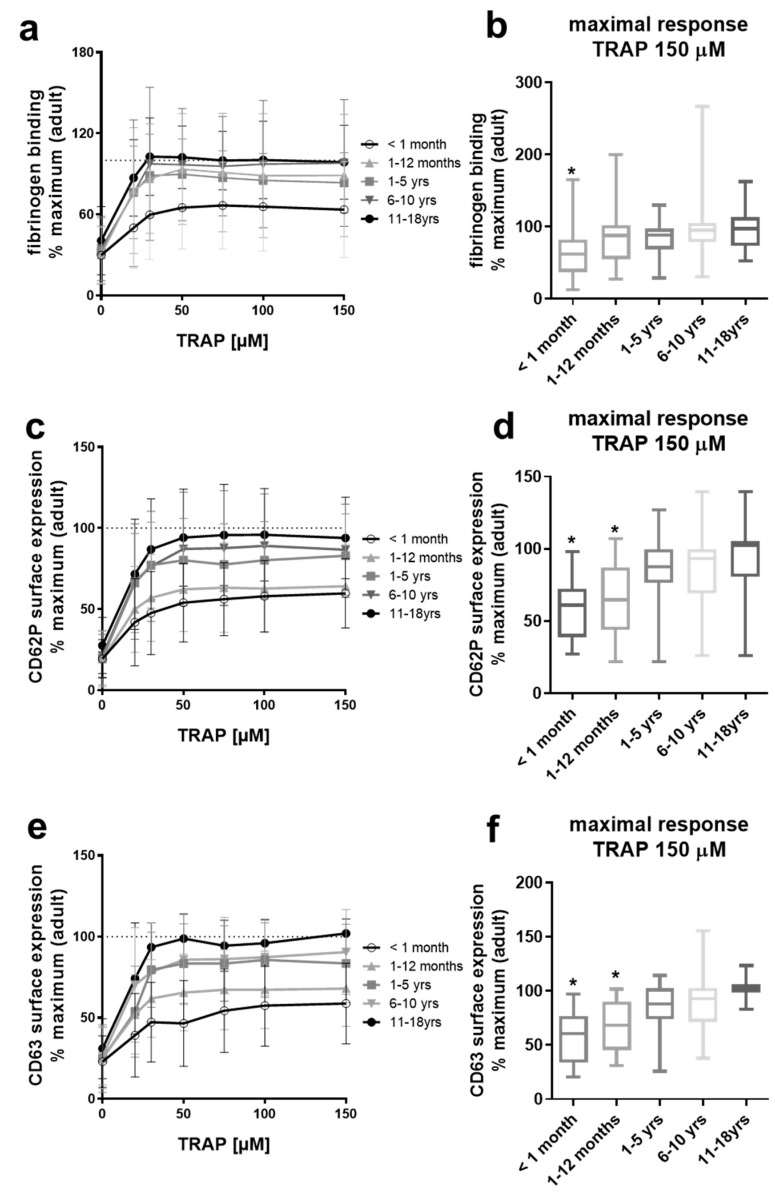
Suppressed platelet responses to TRAP in full-term neonates and infants. Platelet fibrinogen binding (**a**,**b**) and surface expression of CD62P (**c**,**d**) and CD63 (**e**,**f**) in diluted platelet-rich plasma obtained from five pediatric age groups ex vivo and after stimulation with increasing concentrations of the thrombin receptor activating peptide (TRAP). Results are presented as mean ± SD. Data (*n* = 10 per pediatric age group) are normalized to maximal response measured in adult control samples run in parallel (*n* ≥ 3 per run, *n* = 20–25 in total). The maximal responses attained with the highest concentration of TRAP (150 µM) for each age group are depicted in (**b**,**d**,**f**) as medians, with min and max values indicated. * *p* < 0.05 vs. adults.

We first examined age-dependent shifts in platelet activation by TRAP, a hexapeptide (SFFLRN) corresponding to the tethered ligand domain of protease-activated receptor 1 (PAR1) that is exposed upon proteolytic activation by thrombin. We here report that the ability of TRAP to elicit platelet fibrinogen binding was markedly decreased only in neonates (<1 month) independent of the used TRAP concentration, whereas platelets from infants (1–12 months), children (1–5 years; 6–10 years) and adolescents (11–18 years) showed similar fibrinogen binding compared to adults (Figure 1a,b). Interestingly, TRAP-induced expression of CD62P (Figure 1c,d) and CD63 (Figure 1e,f) on the platelet surface was significantly diminished in neonates and infants, with age-dependent normalization toward activation levels seen in adult platelets.

Although platelets from all age groups had normal mean platelet volume (Figure S1a), platelets from neonates and infants/children up to 4 years of age presented reduced levels of the αIIbβ3 integrin, which increased but were still slightly reduced up to 12 years of age (Figure S1b).

### 2.2. Gradual Hypo-Reactivity of Platelets from Pediatric Populations in Response to ADP

**Figure 2:**
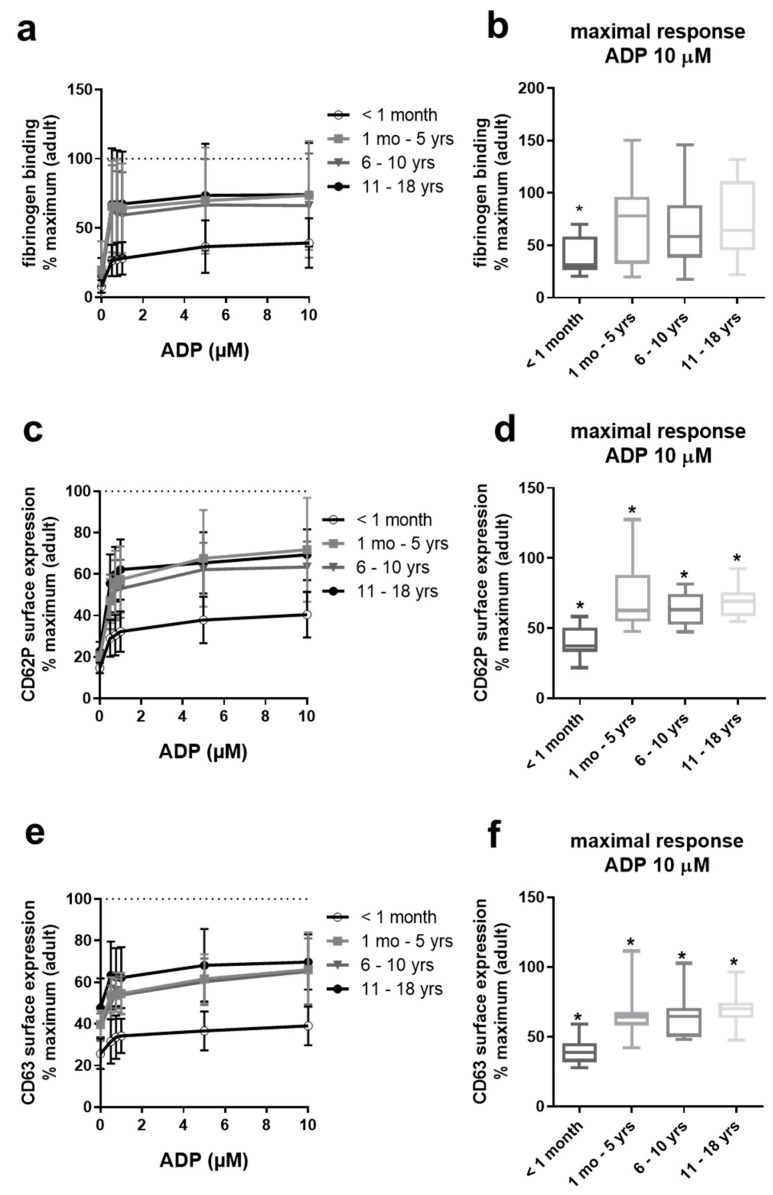
Hypo-reactivity of platelet granule exocytosis in response to ADP of neonates, infants, children, and adolescents. Platelet fibrinogen binding (**a**,**b**) and surface expression of CD62P (**c**,**d**) and CD63 (**e**,**f**) in diluted platelet-rich plasma obtained from four pediatric age groups ex vivo and after stimulation with increasing concentrations of ADP. Results are presented as mean ± SD. Data (*n* = 10 per pediatric age group) are normalized to maximal response measured in adult control samples run in parallel (*n* = ≥1–2 per run, *n* = 10–22 in total). The maximal responses attained with the highest concentration of ADP (10 µM) for each age group are depicted in (**b**,**d**,**f**) as medians, with min and max values indicated. * *p* < 0.05 vs. adults.

Platelet responsiveness to ADP, by contrast, appeared to improve markedly beyond the age of 1 month, with relatively little difference seen among the age groups thereafter (Figure 2). Full adult potential does not appear to be reached even in adolescents aged 11–18, with the sustained impairment in responsiveness to ADP statistically significant for CD62P and CD63 externalization in all age groups. A similar trend in terms of fibrinogen binding was not significant (Figure 2b). The greatest suppression of ADP responses was consistently observed in neonatal platelets, reaching the median below 50% of adult potential at the highest concentration of agonist tested (Figure 2b,d,f).

### 2.3. Collagen-Induced Platelet Activation Is Slightly Reduced in Adolescents, Differentially Reduced in Infants and Children, But Severely Impaired in Neonates

**Figure 3:**
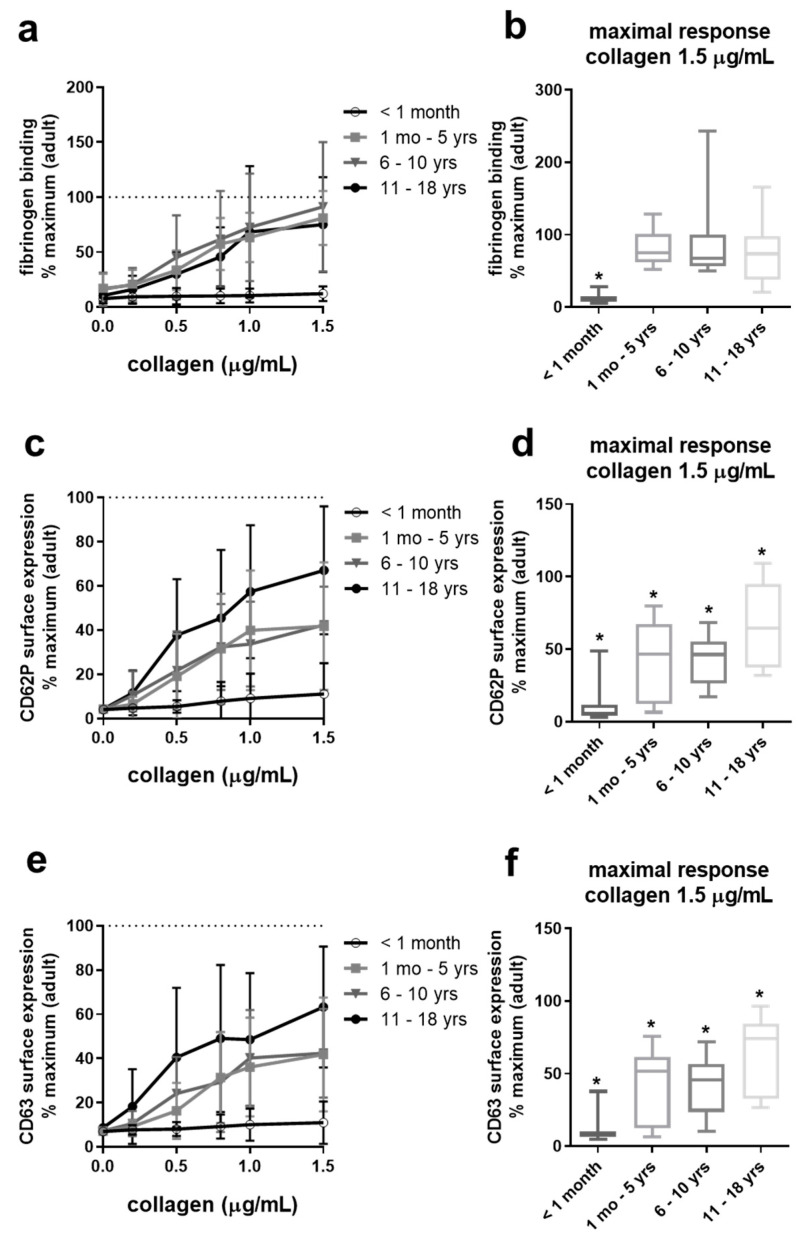
Hypo-reactivity of platelet granule exocytosis in response to collagen of neonates, infants, children, and adolescents. Platelet fibrinogen binding (**a**,**b**) and surface expression of CD62P (**c**,**d**) and CD63 (**e**,**f**) in diluted platelet-rich plasma obtained from four pediatric age groups ex vivo and after stimulation with increasing concentrations of collagen. Results are presented as mean ± SD of *n* = 10. Data (*n* = 10 per pediatric age group) are normalized to maximal response measured in adult control samples run in parallel (*n* = ≥1–2 per run, *n* = 20–23 in total). The maximal responses attained with the highest concentration of collagen (1.5 µg/mL) for each age group are depicted in (**b**,**d**,**f**) as medians, with min and max values indicated. * *p* < 0.05 vs. adults.

Similar to our results of ADP-induced platelet activation, we observed an age-dependent improvement of responsiveness to collagen (Figure 3). Fibrinogen binding to platelets of children ranging from infants to adolescents approached the adult potential (Figure 3a,b). However, CD62P and CD63 surface expression were still markedly diminished on platelets from infants and young children, and even adolescent platelets showed slightly reduced granule secretion compared to adults (Figure 3d,f). Strikingly, platelets from full-term neonates exhibit severely impaired responsiveness to collagen even at high collagen concentrations, reaching the median below 15% of adult potential.

Platelets from neonates and infants/children up to 12 years of age presented slightly but significantly reduced levels of the collagen receptor subunit β1 integrin, with a higher reduction in the group < one year of age (Figure S1c).

### 2.4. Differentially Impaired Responsiveness of Neonatal Platelets to Collagen and Convulxin

**Figure 4:**
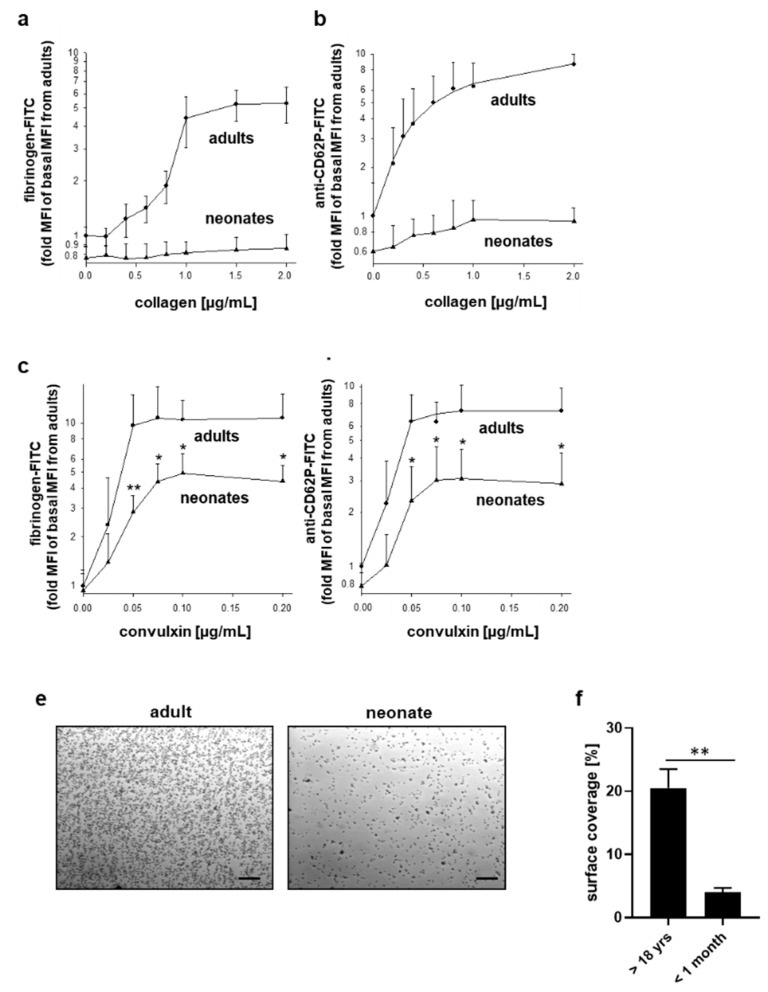
Differential hypo-responsiveness of neonatal platelets in response to collagen and convulxin compared to adults. Platelet fibrinogen binding (**a**) and surface expression of CD62P (**b**) in diluted platelet-rich plasma obtained from full-term neonates ex vivo and after stimulation with increasing concentrations of collagen. Platelet fibrinogen binding (**c**) and surface expression of CD62P (**d**) in diluted platelet-rich plasma obtained from full-term neonates ex vivo and after stimulation with increasing concentrations of convulxin. Platelet adhesion of neonatal and adult platelets to collagen for 3 min at 200 s^−1^ in citrated-whole blood assessed by the cone and plate(let) analyzer; representative May–Grünwald staining of adhered/aggregated platelets (**e**); quantification of adhered platelets expressed as surface coverage in % (**f**). Results are presented as mean ± SD, *n* = 10 per group. * *p* < 0.05; ** *p* < 0.01 vs. adults. Scale bar: 50 µm.

When basal mean fluorescence intensities of fibrinogen-FITC or anti-CD62P antibody binding were normalized to the values determined in adult platelets on the same experimental day, neonatal platelets bound about 20% less fibrinogen and about 40% less anti-CD62P antibody in platelet-rich plasma (PRP) under basal conditions ex vivo (Figure 4a,b). Stimulation with collagen even at a concentration of 2 µg/mL resulted only in a marginal increase in fibrinogen binding and P-selectin surface expression, which were 5 to 8-fold less than observed for maximal activation of adult platelets. Stimulation of neonatal platelets with the GPVI-agonist convulxin was also associated with significant hypo-responsiveness but was not as pronounced as for collagen (Figure 4c,d). Adhesion of neonatal platelets in citrated-whole blood to immobilized collagen at a low shear rate of 200 s^−1^ reached only about 25% of adult platelet surface coverage in the cone and plate(let) analyzer (Figure 4e,f).

Platelets from neonates and infants < one year of age showed normal platelet counts (data not shown), normal MPV (Figure S1a), and normal surface expression of the TSP-1 receptor CD36 (Figure S1e), whereas surface presentation of the αIIbβ3-integrin (Figure S1b) and the β1-subunit (Figure S1c) of the collagen receptor integrin α2β1 was mildly but significantly reduced on neonatal platelets.

A typically reported difference between neonates and adults is the presence of ultra-large high molecular weight VWF multimers in newborns, which possibly compensates for the net platelet hypo-functionality. We confirmed the hyper-responsiveness of neonatal platelets to VWF derived from autologous plasma. VWF-binding to platelets in PRP was about three-fold increased induced by ristocetin compared to adult platelets as assessed by flow cytometry (Figure S2a). In addition, adhesion of neonatal platelets to immobilized VWF of autologous plasma was significantly increased at the arterial shear rate of 1700 s^−1^, with 40% elevated surface coverage compared to adult platelets (Figure S2b,c). Platelet surface levels of GPIbα were normal in neonates and infants < one year of age (Figure S1d).

### 2.5. Neonatal Platelets Exhibit TSP-1 Deficiency and Exogenous TSP-1 Rescues the Platelet Activation Defect in Response to Collagen

**Figure 5:**
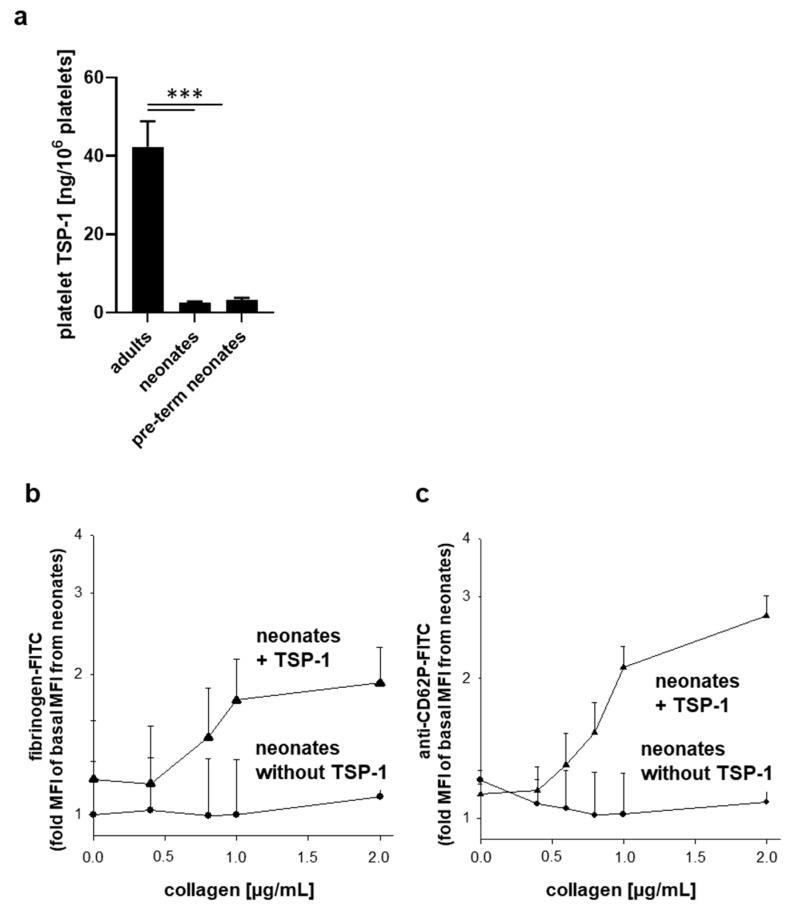
TSP-1-deficiency of neonatal platelets. Platelet TSP-1 content in adults (*n* = 39), full-term (*n* = 34) and pre-term (*n* = 9) (**a**). Platelet fibrinogen binding (**b**) and surface expression of CD62P (**c**) in diluted platelet-rich plasma obtained from full-term neonates ex vivo and after stimulation with increasing concentrations of collagen in the absence or presence of 25 µg/mL added platelet-derived TSP-1. Results are presented as mean ± SD, *n* = 10 per group. *** *p* < 0.001 vs. adults.

The control of VWF size is predominantly attributed to cleavage by ADAMTS13. However, TSP-1 contributes to the control of VWF size due to its disulfide reductase activity [46,47]. Thus, the more TSP-1, the smaller the vWF multimers. Interestingly, in our study, neonatal (both term and pre-term) platelets contained markedly reduced levels of TSP-1 in comparison with adults (Figure 5a).

To examine if the deficiency of TSP-1 in neonatal platelets contributes to their relative activation defect in response to collagen, we incubated diluted PRP from neonates with highly purified TSP-1 of platelets from healthy donors (25 µg/mL) prior to platelet stimulation. Collagen was found to dose-dependently increase both fibrinogen binding (Figure 5b) and externalization of P-selectin (Figure 5c) in neonatal platelet solely in the presence of exogenous platelet-derived TSP-1.

### 2.6. Platelets from Neonates, Infants, Children, and Adolescents Respond Normally to the TSP-1-C-Terminus-Derived Peptide RFYVVMWK

**Figure 6:**
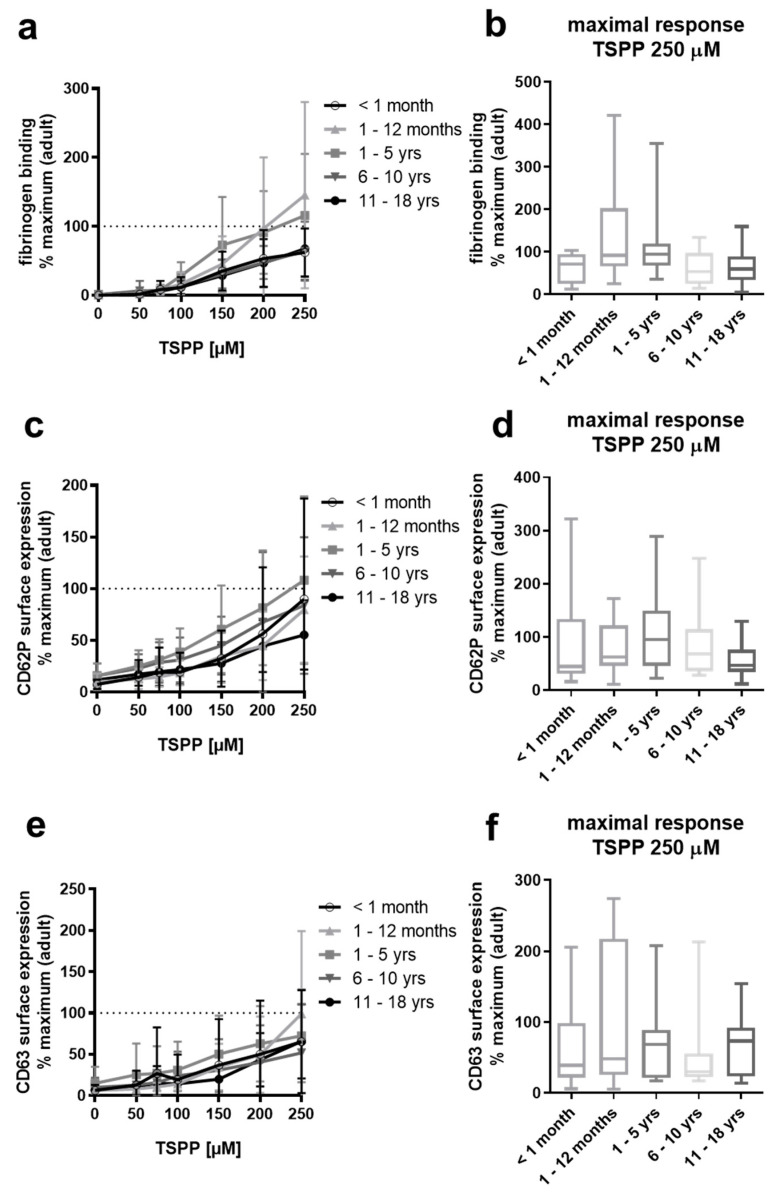
Normal platelet reactivity in response to TSPP of neonates, infants, children, and adolescents. Platelet fibrinogen binding (**a**,**b**) and surface expression of CD62P (**c**,**d**) and CD63 (**e**,**f**) in diluted platelet-rich plasma obtained from four pediatric groups ex vivo and after stimulation with different concentrations of TSPP RFYVVMWK. Results are presented as mean ± SD. Data (*n* = 10 per pediatric age group) are normalized to maximal response measured in adult control samples run in parallel (*n* = ≥3 per run, *n* = 20–25 in total). The maximal responses attained with the highest concentration of TSPP (250 µM) for each age group are depicted in (**b**,**d**,**f**) as medians, with min and max values indicated.

Our observation that supplementation of diluted PRP with platelet-derived TSP-1 restores responsiveness of neonatal platelets to collagen prompted us to test the responsiveness of platelets from our pediatric groups to a peptide corresponding to the RFYVVMWK sequence within the C-terminus of TSP-1 (TSPP), which has been shown to activate platelets via the Fc receptor γ-chain–associated signaling pathway [41]. Interestingly, platelet responsiveness to this TSP-1 peptide did not differ significantly across the age groups compared to adults (Figure 6).

## 3. Discussion

The reactivity of platelets is developmentally regulated, and it is well established that platelets from neonates are markedly hypo-responsive to several physiological agonists [8]. Although altered expression of distinct surface receptors and receptor-coupled signaling have been described, the underlying mechanisms responsible for platelet dysfunction in neonates are incompletely understood. In addition, there are still controversial reports at which age platelets from pediatric populations reach comparable functions of adults [17,21,48].

In this study, we used flow cytometry to establish reactivity ranges of platelets in PRP from up to five pediatric groups for the agonists TRAP, ADP, and collagen, normalized to the maximal response obtained with adult control platelets measured in parallel. Flow cytometry offers several advantages for the analysis of platelet (dys)function from pediatric populations compared to platelet aggregation tests [44,45,49,50]. (1) Small blood volumes, e.g., 1.3 mL, are sufficient to analyze platelet function induced by different agonists and receptor presentation in whole blood or diluted PRP. (2) Flow cytometry allows the measurement of multiple activation readouts of individual/single platelets. (3) Distinct platelet populations can be discriminated against in relation to the platelet activation status and donor and autologous platelets after platelet transfusion therapy [51]. (4) Platelet-platelet contact is not required as functional parameters are monitored on a single platelet. This enables platelet function analysis in thrombocytopenic samples, such as immune thrombocytopenia and combined platelet function and number disorders [52]. Our data clearly demonstrate that age-dependent changes of platelet reactivity and the achievement of adult platelet function depend on the agonist used and the platelet activation parameter.

The binding of soluble fibrinogen to activated αIIbβ3 integrin on stimulated platelets is a prerequisite for platelet aggregation. We used increasing concentrations of TRAP to stimulate the G protein-coupled thrombin receptor PAR1. Our data revealed a dose-dependent increase in platelet binding of exogenous FITC-coupled fibrinogen for all age groups, which was significantly diminished only for full-term neonates (<1 month) compared to adults (>18 years). From these data, we conclude that platelets from infants (≥1 month–12 months) show comparable reactivity as adult platelets in terms of fibrinogen binding in response to TRAP. We observed a significant, of approximately 30%, lower abundance of integrin αIIbβ3 on the platelet surface of neonates, in keeping with other studies [31,53,54] and infants and toddlers up to 4 years of age, suggesting that a modestly reduced level of αIIbβ3 at the platelet surface is not crucially responsible for decreased platelet binding of fibrinogen. Indeed, lower levels of PAR receptors, which have been shown for neonatal platelets to cause hypo-aggregability in response to thrombin [55], might serve as one explanation. Interestingly, TRAP-induced exocytosis of platelet α-granules, δ-granules, and lysosomes, associated with the release of important autocrine and paracrine platelet activators and modulators, was markedly diminished in neonates as reported [20,53,54] and still significantly decreased in infants. A study by Hézard et al. even showed significantly decreased αIIbβ3 surface levels and hypo-responsiveness, i.e., TRAP-induced binding of autologous plasma fibrinogen and P-selectin surface expression, for platelets from pediatric groups up to 15 years of age when the analysis was performed in citrated-whole blood by flow cytometry [21].

In our study, ADP stimulation elicited a lower (approximately 30%) degree of fibrinogen binding to neonatal platelets compared to platelets from other pediatric groups, which approached adult potential. Baker-Groberg et al., as well as Hardy et al., also reported reduced ADP-mediated αIIbβ3 activation of neonatal platelets, measured with PAC-1 antibody [30] and fibrinogen binding [31] of neonatal platelets in whole blood; however, this reported hypo-responsiveness was less pronounced. In comparison, we found that platelet granule exocytosis was not only impaired in neonates in response to ADP but was also significantly reduced in the other pediatric age groups.

For collagen, we observed a nearly complete loss of platelet reactivity in neonates, whereas platelets from other pediatric groups (≥1 month–18 years) were able to show adult-like fibrinogen binding but were also hypo-reactive in an age-dependent manner in terms of granule exocytosis. Thus, our flow cytometry data revealed the need to establish detailed age-specific reference ranges of pediatric populations, especially for different platelet activation responses such as integrin αIIbβ3 activation and degranulation.

Hardy et al. reported mildly reduced expression of GPVI associated with reduced downstream signaling via Syk, platelet fibrinogen binding, and P-selectin surface expression of full-term neonatal platelets in response to the GPVI-agonist collagen-related peptide [31]. Using another GPVI agonist, convulxin, we confirmed a significant reduction in platelet fibrinogen binding and P-selectin surface expression of full-term neonates compared to adults. However, this suppression was less pronounced than observed for collagen-stimulated platelets, suggesting that the collagen-dependent defect of neonatal platelets cannot solely be explained by GPVI-coupled signaling. Unlike the study by Hardy et al., our data indicate mildly but significantly reduced levels of the collagen integrin α2β1. Furthermore, neonatal platelets showed about 75% decreased adhesion to collagen at low shear compared to adult platelets. This observation contrasts with data reported by Baker-Groberg et al., who detected similar adhesion of neonatal platelets under static and low flow conditions compared to adults [30]. However, these authors analyzed the blood of only three neonates and only for 30 s of flow associated with less than 10% platelet surface coverage, which might explain the different results. We validated our experimental settings for flow cytometry and flow-based assay/cone and plate(let) analyzer by investigating responsiveness of neonatal platelets dependent on unusually large multimeric VWF in neonatal plasma/blood, which has been previously described to contribute to neonatal platelet hyper-responsiveness [32,36,56]. Our data demonstrated up to a three-fold increase in binding of neonatal VWF to the platelet surface induced by ristocetin as well as up to 40% increase in surface coverage of neonatal platelets on neonatal VWF under arterial shear conditions, supporting hyper-reactivity of neonatal platelets to neonatal VWF.

We provide the first potential mechanistic link between the hypo-responsiveness of neonatal platelets to collagen on the one hand and the VWF-mediated hyper-reactivity on the other. Our data reveals that platelets from pre-term and full-term neonates exhibit pronounced deficiency in TSP-1. Interestingly, near loss of collagen-induced fibrinogen binding and P-selectin surface expression observed for neonatal platelets could be induced and restored in the presence of platelet-derived TSP-1. Of note, TSP-1 alone in the absence of collagen did not induce platelet activation. It has been reported that TSP-1 promotes collagen-induced aggregation of human platelets through inhibition of cyclic adenosine monophosphate (cAMP)/protein kinase A (PKA) signaling via CD36 [38]. TSP-1 and CD36 have also been implicated in thrombus stabilization on immobilized collagen underflow at low shear in mice [57]. As we found no differences in surface expression levels of CD36 on platelets from neonates compared to adolescents and adults, binding of TSP-1 to CD36 might control collagen-dependent platelet activation, which is strongly impaired in neonates. However, Kuijpers et al. observed changes in thrombus stabilization but not in thrombus formation at low shear in vitro from TSP-1- or CD36-deficient mice, suggesting that TSP-1 might not essentially be involved in platelet adhesion to collagen at low shear. Future studies need to clarify if TSP-1 deficiency contributes to impaired adhesion of neonatal platelets to collagen at low shear.

From our data, it is likely that slightly but significantly reduced surface levels of the collagen receptors integrin α2β1, as shown by us, and GPVI [31] as well as related impaired signaling contributes rather to hypo-adhesive properties of neonatal platelets than TSP-1 deficiency. In addition, we observed normal activation of platelets from neonates and from all other pediatric age groups induced by the peptide RFYVVMWK based on the C-terminal cell-binding domain of TSP-1. This peptide has been shown to mediate platelet agglutination via binding to platelet CD47 [40] and platelet activation via FcRγ-chain-dependent signaling [41]. However, the responsible TSP-1 receptor(s) and receptor-coupled signaling, which are involved in controlling collagen-dependent platelet activation and TSP-1-dependent signaling, which is impaired in neonatal platelets, have to be resolved in future studies. On the other hand, TSP-1 controls the size of VWF multimers through its enzymatical protein disulfide reductase activity [47]. Therefore, TSP-1 could locally contribute to the reduction in VWF multimers, whereas in the blood of neonates, high levels of unusually large VWF may be partially explained by TSP-1-deficiency in platelets. However, this potential causal relationship has to be addressed in the future.

So far, it is not known which hemostatic alterations during development were the ontogenetic drivers. There is evidence that TSP-1 is an important regulator of innate immunity, which is under developmental control [58,59,60]. Therefore, it could be speculated that platelet hypo-responsiveness in neonates represents an immature state that is improved with maturation and aging. It has been shown that also neonatal and fetal mouse platelets are hypo-reactive, especially in response to ITAM-related platelet receptor activation via GPVI and CLEC-2 and in response to thrombin, respectively [31,61]. However, further studies have to identify further mechanistic insights of similarities and differences in neonatal platelet hypo-reactivity between humans and mice and whether important age-related determinants of platelet hyper-reactivity, e.g., redox homeostasis, inflammatory state, or energy metabolism [62,63], also affect developmentally regulated platelet reactivity.

In conclusion, our data suggest that TSP-1 might serve as a balancer of collagen- and VWF-dependent platelet function. In healthy neonates, the loss of TSP-1 in platelets contributes to hypo-responsiveness to collagen, which is counterbalanced by hyper-responsiveness to high molecular VWF to prevent bleeding. Furthermore, our results implicate that the flow cytometric evaluation of platelet function, especially from neonates and infants, is only valid in comparison to age-specific reference ranges. The comparison to reference ranges from adults might lead to misdiagnosis of several platelet function defects in healthy children. However, the laboratory establishment of such reference values is not usually practicable for clinical routine laboratories and is limited by ethical issues.

## 4. Materials and Methods

### 4.1. Materials

Human fibrinogen (Enzyme Research Laboratories, South Bend, IN, USA) was conjugated with fluorescein iso-thiocyanate (FITC) via FITC-celite (Sigma-Aldrich, St Louis, MO, USA) as described [28]. Soluble type I collagen (kindly provided by J. Rautersberg, University of Münster, Münster, Germany) was prepared for flow cytometry experiments as described [28]. Human TSP-1 was purified from human platelets as described [64,65]. The purified TSP-1 presented with a single band of about 180 kDa after SDS-PAGE under reduced conditions and sensitive silver staining without any detected contamination (Figure S3). FITC-conjugated monoclonal anti-CD62P, anti-CD63, and control antibodies were from Becton Dickinson (Heidelberg, Germany). FITC-conjugated monoclonal anti-CD42b (clone SZ2), anti-CD41/CD61 (clone P2), anti-CD29 (clone K20) and anti-CD36 (clone FA.152) antibodies were purchased from Beckman Coulter (Krefeld, Germany). FITC-conjugated polyclonal anti-VWF antibody was from Bio-Rad (Puchheim, Germany). The C-terminal thrombospondin peptide RFYVVMWK (TSPP) were from Bachem Biochemica GmbH (Weil am Rhein, Germany), ADP and bovine serum albumin were from Sigma-Aldrich (Munich, Germany). The thrombin receptor activating peptide TRAP-6 with the amino acid sequence SFLLRN was purchased from Nova Biochem (via Merck Chemicals, Darmstadt, Germany). Ristocetin was from American Biochemical and Pharmaceuticals Ltd. (London, UK). Quantum™ Simply Cellular^®^ anti-mouse IgG was from Bangs-Laboratories (Fishers, IN, USA). Precision Plus protein standards for SDS-PAGE were from Bio-Rad Laboratories (Feldkirchen, Germany).

### 4.2. Study Participants and Blood Collection

Citrate-anticoagulated blood collection from healthy Caucasian sex-matched pediatric subjects up to 18 years of age (Table 1) with no history or family history of thrombosis or bleeding episodes was performed by trained pediatricians during routine visits with the informed consent of the parents or legal guardian. Older children (≥12 years) were also asked to provide informed consent. Volumes of peripheral venous blood were 1.3 mL from children under 5 years of age, and older children and adolescents provided 3 mL of blood into appropriate 3.2% citrate S-Monovettes (Sarstedt, Nümbrecht, Germany), which are sufficient for the analysis of platelet function and receptor presentation by flow cytometry.

Children and adults with underlying diseases such as cardiac malformation, metabolic, hematologic-oncologic underlying disorders, with a family history of thrombosis or bleeding episode (core family) were excluded. Furthermore, study participants must prove a normal platelet count, normal hematocrit and hemoglobin, normal prothrombin time and aPTT, normal plasma levels of VWF antigen and fibrinogen. Blood from healthy adult donors (>18 years of age), who did not take any drugs affecting platelet function for at least 14 days prior to analysis, was obtained with informed consent and measured in parallel with juvenile samples. Blood samples were immediately transported at room temperature, avoiding delay, and shaking to limit pre-activation. The studies were approved by the local Ethics Committee of the University of Münster (“Platelet function analysis in neonates, children (0–17 years of age) and adolescents”, 1998-02-14) and of the University Medical Center Mainz (Study No. 837.302.12;25.07.12; 2018-13290_1;27.07.2018) and performed in accordance with the Declaration of Helsinki.

### 4.3. Preparation of Platelet-Rich Plasma and Washed Platelets

Platelet-rich plasma (PRP) was prepared by differential centrifugation at 200× *g* for 10 min at room temperature shortly after the blood was taken [67]. Platelets in PRP were washed as previously described [68]. Briefly, 1/10 volume of CTADX10 (citric acid 0.11 M, theophylline 15 mM, adenosine 3.7 mM, and dipyridamole 0.198 mM, pH 5.0) was added to the PRP followed by centrifugation at 800× *g* for 10 min at room temperature. The pelleted platelets were resuspended in saline containing ACD (citric acid 71 mM, sodium citrate 0.085 mM, d-glucose 11 mM), centrifuged and resuspended in HEPES buffer (NaCO_3_ 12 mM, NaCl 138 mM, d-glucose 5.5 mM, KCl 2.9 mM, HEPES 50 mM, CaCl_2_ 1 mM, MgCl_2_ 2 mM, pH 7.2).

### 4.4. Flow Cytometric Analysis of Platelet Surface Receptors

Formaldehyde-fixed platelets in diluted PRP were incubated with monoclonal anti-αIIbβ3 (CD41/CD61)-FITC, anti-GPIbα (CD42b)-FITC, anti-GPIX-FITC, anti-integrin-β1 (CD29)-FITC, anti-GPIV (CD36)-FITC antibodies and mouse IgG isotype as described [69,70]. Absolute numbers of antigen-binding sites (ABS) per platelet were calculated using Quantum™ Simply Cellular^®^ anti-mouse IgG according to the manufacturer’s instructions. The FACScan (Becton Dickinson, Heidelberg, Germany) was used in a standard configuration with 488 nm excitation wavelength and a 530 nm bandpass filter. Platelets were gated, and data were obtained from fluorescence channels in a logarithmic model. Mean fluorescence intensities (MFI) were then linearized. A total of 5000 events were analyzed for each data point. Readings were corrected for background binding determined in parallel samples with FITC-conjugated isotype-specific mouse IgG.

### 4.5. Flow Cytometric Analysis of Agonist-Induced Platelet Activation

Depending on sample volume and concentration, PRP was adjusted to 2.5 or 5 × 10^7^ platelets/mL with PBS, pH 7.4. Diluted PRP was stimulated with different agonists for 3 min at room temperature. The reaction was stopped by the addition of formaldehyde (final concentration 0.5%) and fixed at room temperature for 30 min. Samples were washed with PBS pH 7.4, centrifuged (800× *g*, 10 min, room temperature), and resuspended in 100 µL PBS. FITC-coupled antibodies against CD62P, CD63, and VWF were added at saturating concentrations followed by 1 h incubation at room temperature in the dark. For determination of fibrinogen binding, PRP was preincubated for 3 min at room temperature with 150 μg/mL fibrinogen-FITC prior to agonist stimulation. Fixed and labeled samples were washed, resuspended in 500 μL PBS, and analyzed by flow cytometry as described [67].

### 4.6. Platelet Adhesion Analysis under Flow

Platelet adhesion experiments were performed in the cone and plate(let) analyzer as described [37]. Polystyrene wells of four-well culture plates (Nunc, Wiesbaden, Germany), coated with 15 µg/mL of type 1 collagen and subsequently blocked with 3% bovine serum albumin (BSA), or four-well polystyrene plates, which enables the analysis of platelet adhesion to autologous plasma VWF [71], were used.

### 4.7. Quantification of Platelet TSP-1

TSP-1 was quantified in lysates of washed platelets from adults, neonates, and pre-term neonates by an enzyme-linked immunosorbent assay according to the manufacturer’s instructions (ab193716, Abcam, Cambridge, UK).

### 4.8. SDS-PAGE and Protein Silver Staining

Isolated human TSP-1 was subjected to SDS-PAGE (7.5% polyacrylamide) under reducing conditions in Laemmli buffer after boiling at 95 °C for 10 min. Separated proteins were visualized by silver staining, according to Blum et al. [72].

### 4.9. Data Analysis and Statistical Methods

Data are shown as mean ± standard deviation (SD) when data were normally distributed or as median (range) as indicated. Some data sets (Figure 1, Figure 2, Figure 3 and Figure 6) were normalized to the maximal platelet response obtained in adult control samples (set as 100%) measured in parallel (median of ≥3 adult donors). Some datasets (Figure 4a–d, Figure 5b,c, and Figure S3a) were normalized to the basal platelet response (set as 1) obtained in control samples from adults or neonates. For statistical analysis, GraphPad Prism software (version 6.07 for Windows, GraphPad Software, La Jolla, CA, USA) was used. In the case of normal distribution and equal variance, parametric tests were performed for comparisons between two groups by two-tailed Student’s *t*-test, for comparisons between >2 groups by one-way analysis of variance (ANOVA) followed by Tukey’s multiple comparisons test. For comparisons of each number of groups with a single control group, one-way ANOVA followed by Dunnett post hoc test was performed. For comparison of nonparametric data sets, the Mann–Whitney *U* test (comparison of 2 groups) or the Kruskal–Wallis test (comparison of >2 groups) followed by Dunn post hoc test for multiple comparisons was used. *p* < 0.05 was considered statistically significant.

## Figures and Tables

**Table 1:** Pediatric age categories according to WHO [66].

Neonates (Full-Term ≥37 Weeks’ Gestation)	<1 Month (Total *n* = 46)
Infants	1–12 months (total *n* = 51)
Young children	1–5 years (total *n* = 56)
Children	6–10 years (total *n* = 47)
Adolescents	11–18 years (total *n* = 52)

## Data Availability

The data presented in this study are available on request from the corresponding authors.

## Supplementary Materials

The following are available online at https://www.mdpi.com/article/10.3390/ijms22094883/s1, Figure S1: Mean platelet volume and platelet surface expression of GPIbα, integrin αIIbβ3, integrin β1 subunit, and CD36 from neonates, infants, children and adolescents, Figure S2: VWF-mediated hyper-responsiveness of neonatal platelets compared to adults, Figure S3: Analysis of highly purified human TSP-1 from peripheral blood by SDS-PAGE.
