# Supplementary material for: Age-Dependent Control of Collagen-Dependent Platelet Responses by Thrombospondin-1—Comparative Analysis of Platelets from Neonates, Children, Adolescents, and Adults
Source: Int J Mol Sci. 2021 May 5;22(9):4883. doi: 10.3390/ijms22094883 (PMC8124951; doi:10.3390/ijms22094883)
Supplement: Supplementary file 1 [file ijms-22-04883-s001.zip › ijms-1197410-supplementary.pdf]

International Journal of
Molecular Sciences

MDPI

Supplementary Material

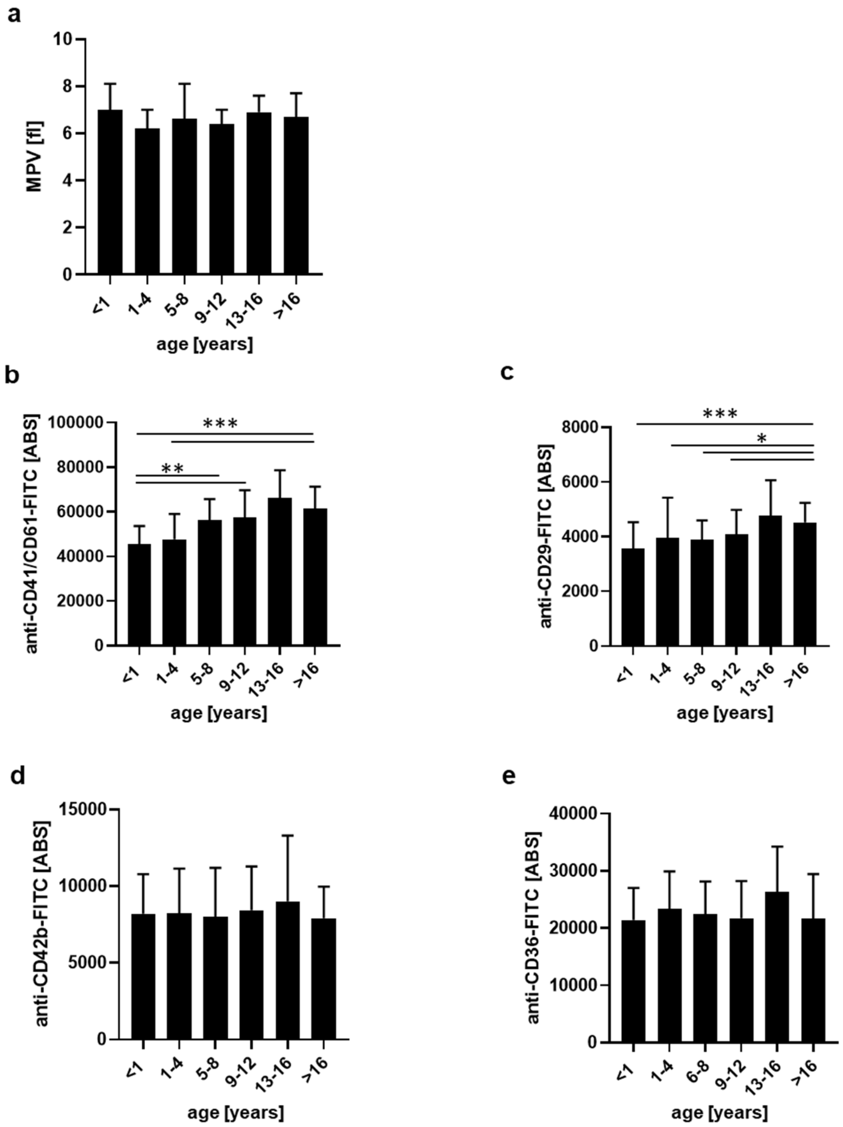

Figure S1. Mean platelet volume and platelet surface expression of GPIbα, integrin αIIbβ3, integrin β1 subunit and CD36 from neonates, infants, children and adolescents. (**a**) Mean platelet volume (MPV), n=11-21 per group. Antigen binding sites (ABS) of anti-CD41/CD61-FITC antibody (**b**, n=29-30 per group), anti-CD29-FITC (**c**, n=30 per group), anti-CD42b-FITC antibody (**d**, n=29 per group), anti-CD36-FITC (**e**, n=30 per group). Results are presented as mean ± SD. *P < 0.05; **P < 0.01; ***P < 0.001.

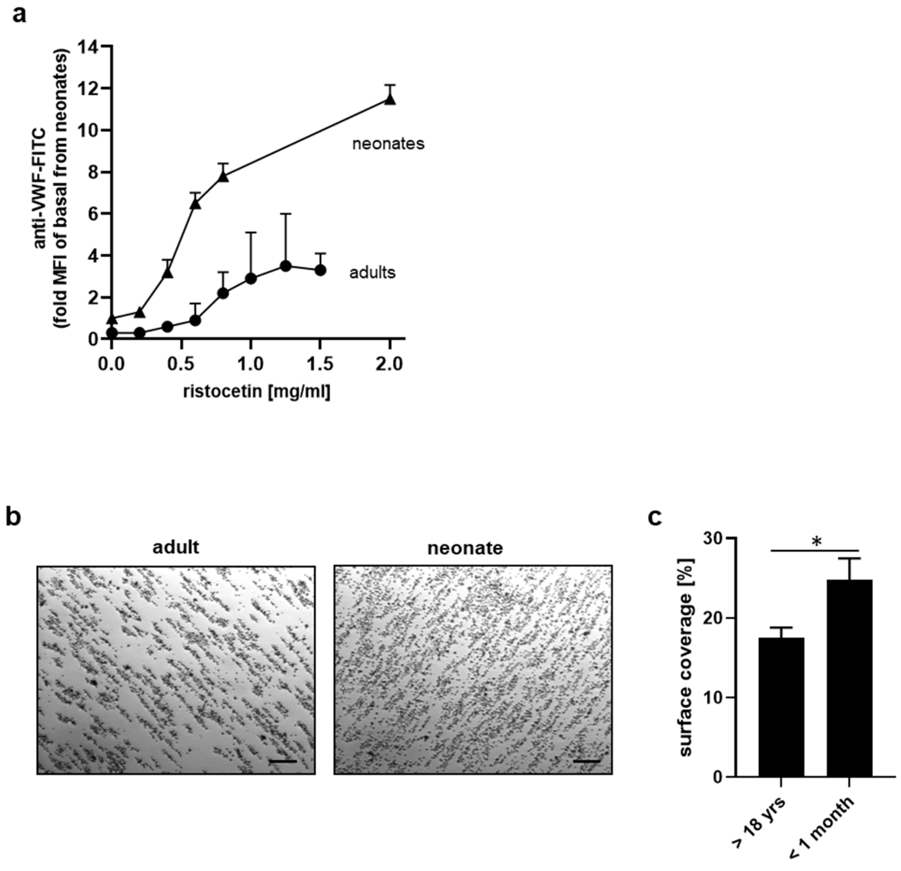

Figure S2. VWF-mediated hyper-responsiveness of neonatal platelets compared to adults. (**a**) Ristocetin-induced platelet VWF-binding in diluted platelet-rich plasma obtained from full-term neonates ex vivo and after treatment with increasing concentrations of ristocetin. Platelet adhesion of neonatal and adult platelets to autologous plasma VWF for 3 min at 1300 s^{-1} in citrated whole blood assessed by the cone and plate(elt) analyser; representative May-Grünwald staining of adhered/aggregated platelets (**b**); quantification of adhered platelets expressed as surface coverage in % (**c**). Results are presented as mean ± SD, n=10 per group. *P < 0.05 vs. adults. Scale bar: 50 μm.

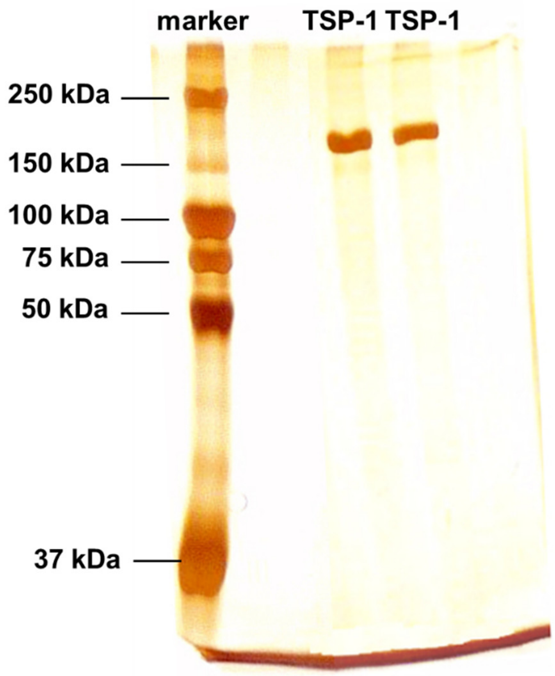

Figure S3. Analysis of highly purified human TSP-1 from peripheral blood by SDS-PAGE. Isolated human TSP-1 (30 µg/lane) was separated by SDS-PAGE (7.5% polyacrylamide) under reducing conditions. Sensitive silver staining demonstrates the high purity of TSP-1, which is separated as single band with an apparent molecular weight of about 180 kDa under reducing conditions.
